# Supplementary material for: Time Perspective Latent Profile Analysis and Its Meaning for School Burnout, Depression, and Family Acceptance in Adolescents
Source: Int J Environ Res Public Health. 2023 Apr 7;20(8):5433. doi: 10.3390/ijerph20085433 (PMC10138429; doi:10.3390/ijerph20085433)
Supplement: Supplementary file 1 [file ijerph-20-05433-s001.zip › ijerph-2174755-supplementary.pdf]

Supplementary Materials

Table S1. Descriptive statistics for measured psychological characteristics (N = 668).

Variable	Min.	Max.	Mean	SD	Skewness	Kurtosis
PFA	1	5	4.39	**0.77**	**−1.47**	**1.61**
LIS	6	30	15.54	**4.56**	**0.56**	**0.21**
BFS	6	29	15.92	4.14	0.47	0.03
BFF	5	25	13.09	4.31	0.34	−0.29
BFH	6	25	14.78	3.94	0.35	−0.36
BFTA	4	20	10.48	3.19	0.69	0.12
NRTF	4	20	11.57	3.43	0.32	−0.60
FIS	4	20	12.47	3.38	0.20	−0.42
SSBS	56	154	93.85	18.46	0.66	0.31
KADS	0	18	5.32	4.48	0.87	0.19
PP	13.0	44.0	28.93	5.46	−0.20	−0.14
PN	11.0	50.0	32.91	7.32	−0.01	−0.50
PF	12.0	43.0	26.41	4.89	0.03	0.46
PH	24.0	75.0	52.58	7.95	−0.19	42
F	14.0	64.0	39.12	8.01	0.20	0.05

Note: Positive Temporal Perspective (PP), Past-Negative Temporal Perspective (PN), Present Fatalist Temporal Perspective (PF), Present-Hedonistic Temporal Perspective (PH) and Future Time Perspective (F); Perceived Family Acceptance (PFA); School burnout (SSBS); Burnout from Studying (BFS); Burnout from Family (BFF); Loss of Interest in School (LIS); Burnout from Homework (BFH); Burnout from Teacher Attitudes (BFTA); Need to Rest and Time for Fun (NRTF); Feeling of Insufficiency at School (FIS); Adolescent Depression (KADS).

Table S2. Boot-strapped likelihood ratio test results (BLRT) **for** time perspective and school burnout profiles.

G	**Time Perspective**		**Student Burnout**		**Time Perspective & Student Burnout**	
	BLRT	***p*-Value**	***LRTS***	***p*-Value**	***LRTS***	***p*-Value**
1 vs. 2	87.67	0.001	1202.62	<0.001	293.22	<0.001
2 vs. 3	93.32	0.001	299.82	<0.001	120.00	<0.001 *
3 vs. 4	155.86	0.001	152.71	<0.001	---	---
4 vs. 5	37.86	0.035	59.42	<0.001	---	---
5 vs. 6	−69.39	0.977	255.62	<0.001	---	---
6 vs. 7	---	---	68.34	<0.001	---	---
7 vs. 8	---	---	17.08	0.996	---	---

Note: *—by the *G* values higher than 3 the algorithm did not converge, so the highest possible value (*G* = 3) was chosen.

Table S3. Differences between pre-and post-COVID 19 samples among five profiles in PFA, SSBS, and KADS scores.

Variable		Profile 1 Mean ± SD	Profile 2 Mean ± SD	Profile 3 Mean ± SD	Profile 4 Mean ± SD	Profile 5 Mean ± SD
Perceived Family Acceptance (PFA)	Pre-COVID 19	4.52 ± 0.04	4.56 ± 0.05	4.45 ± 0.06	5.00 ± 0.00	4.00 ± 0.00
	Post-COVID 19	4.33 ± 0.08	4.23 ± 0.12	3.84 ± 0.16	4.77 ± 0.08	4.20 ± 0.22
	Cohen d	3.00	3.59	5.05	4.07	1.29
	Effect size r	0.83	0.87	0.93	0.90	0.54
Student school burnout (SSBS)	Pre-COVID 19	88.38 ± 0.92	82.72 ± 1.29	90.77 ± 1.54	78.33±12.99	91.50 ± 0.50
	Post-COVID 19	98.05 ± 1.59	96.68 ± 2.29	110.56±2.97	93.62 ± 3.50	128.30±2.30
	Cohen d	7.44	7.51	8.37	1.61	22.11
	Effect size r	0.97	0.97	0.97	0.63	0.99
Adolescent Depression (KADS)	Pre-COVID 19	5.08 ± 0.30	4.80 ± 0.44	6.03 ± 0.53	0.67 ± 0.67	4.50 ± 1.50
	Post-COVID 19	4.34 ± 0.34	4.83 ± 0.54	10.16 ± 0.71	2.77 ± 0.37	8.60 ± 0.63
	Cohen d	2.31	0.06	6.59	3.88	3.56
	Effect size r	0.76	0.03	0.96	0.89	0.87

Note: Pre-COVID 19—students examined before pandemic COVID-19 outbreak; Post-COVID 19—students examined after pandemic COVID 19 outbreak.

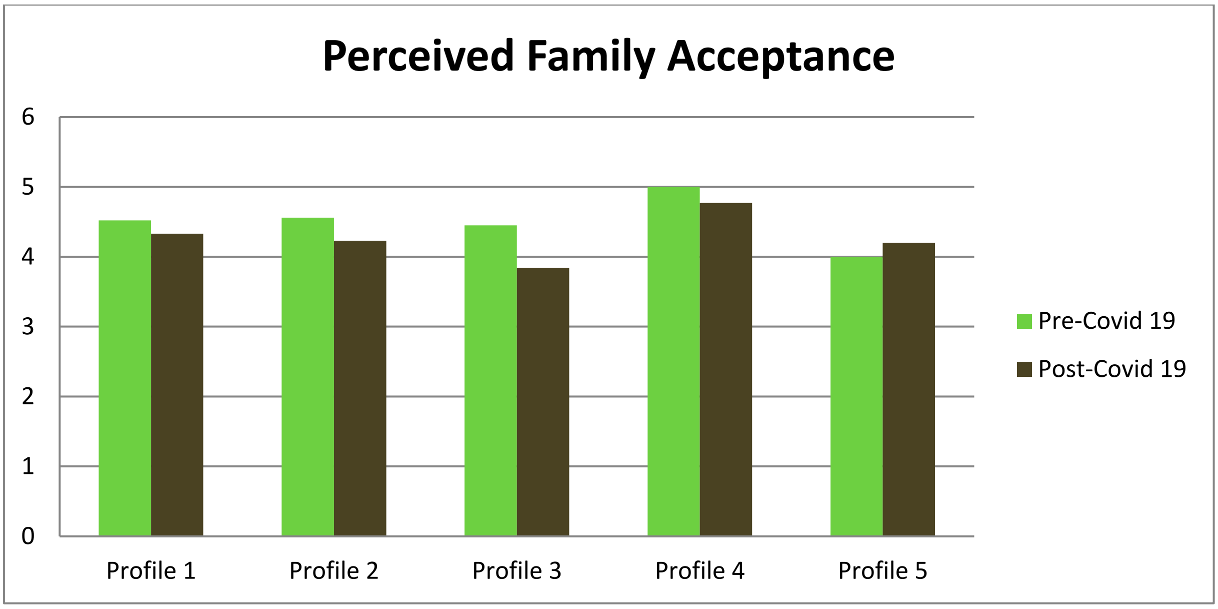

Figure S1. Differences in the levels of perceived family acceptance between pre-and post-COVID 19 samples among five distinguished profiles.

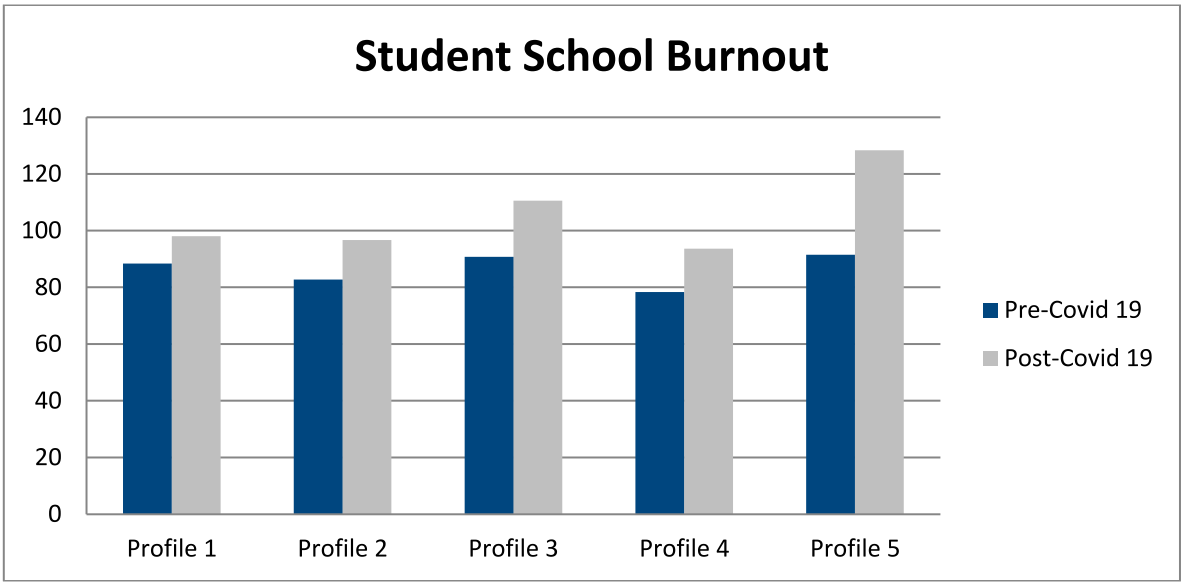

Figure S2. Differences in the levels of student school burnout between pre-and post-COVID 19 samples among five distinguished profiles.

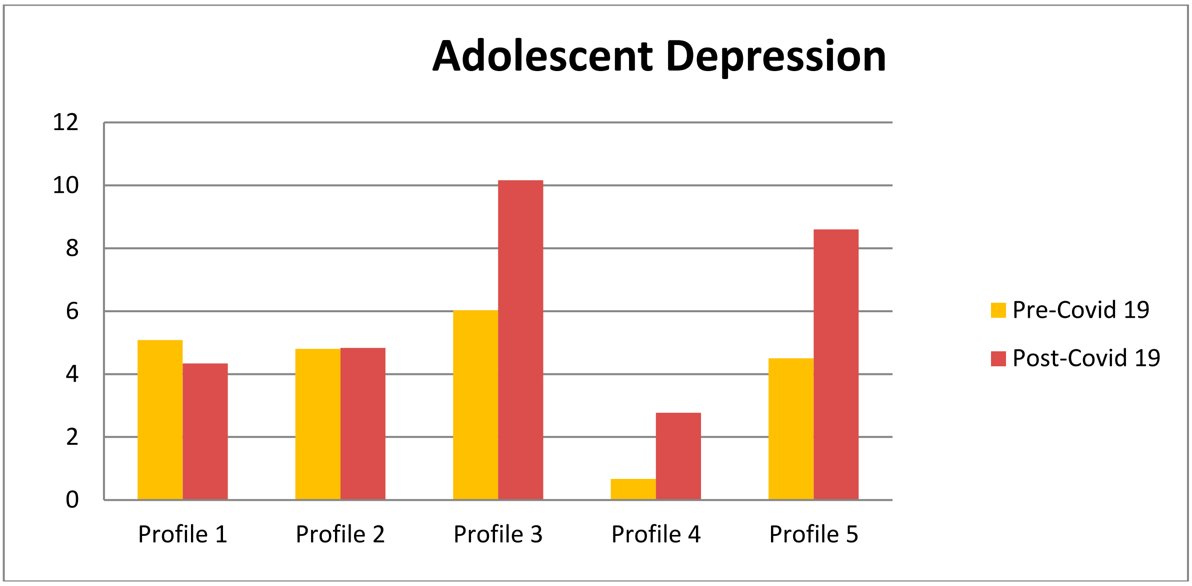

Figure S3. Differences in the levels of adolescent depression between pre-and post-COVID 19 samples among five distinguished profiles.
